# RNAi-Based Suppressor Screens Reveal Genetic Interactions Between the CRL2^LRR-1^ E3-Ligase and the DNA Replication Machinery in *Caenorhabditis elegans*

**DOI:** 10.1534/g3.116.033043

**Published:** 2016-08-18

**Authors:** Batool Ossareh-Nazari, Anthi Katsiarimpa, Jorge Merlet, Lionel Pintard

**Affiliations:** Jacques Monod Institute, Paris-Diderot University, Centre National de la Recherche Scientifique, 75013 Paris, France

**Keywords:** Cullin-RING E3-Ligases, DNA replication, *C. elegans*, Mutant Screen Report

## Abstract

Cullin-RING E3-Ligases (CRLs), the largest family of E3 ubiquitin-Ligases, regulate diverse cellular processes by promoting ubiquitination of target proteins. The evolutionarily conserved Leucine Rich Repeat protein 1 (LRR-1) is a substrate-recognition subunit of a CRL2^LRR-1^ E3-ligase. Here we provide genetic evidence supporting a role of this E3-enzyme in the maintenance of DNA replication integrity in *Caenorhabditis elegans*. Through RNAi-based suppressor screens of *lrr-1(0)* and *cul-2(or209*ts*)* mutants, we identified two genes encoding components of the GINS complex, which is part of the Cdc45-MCM-GINS (CMG) replicative helicase, as well as CDC-7 and MUS-101, which drives the assembly of the CMG helicase during DNA replication. In addition, we identified the core components of the ATR/ATL-1 DNA replication checkpoint pathway (MUS-101, ATL-1, CLSP-1, CHK-1). These results suggest that the CRL2^LRR-1^ E3-ligase acts to modify or degrade factor(s) that would otherwise misregulate the replisome, eventually leading to the activation of the DNA replication checkpoint.

DNA replication is a tightly regulated multistep process that requires the sequential action of several protein complexes that select DNA replication origins, recruit on these origins the DNA replication fork helicase that once activated, unwinds and duplicates the DNA. These events must be tightly coupled to cell cycle progression to ensure that DNA replication occurs once and only once per cell cycle.

DNA replication is thus temporally separated into two steps that are controlled by Cyclin-Dependent Kinase (CDK) activity. The first step, which occurs in mitosis and during the G1 phase of the cell cycle, when Cdk activity is low, involves the loading of a double hexameric Mcm2-7 (minichromosome maintenance 2-7) complex on the chromatin as part of the prereplicative complex (pre-RC) (Evrin *et al.* 2009; Remus *et al.* 2009; Gambus *et al.* 2011; Deegan and Diffley 2016). Pre-RC formation requires several loading factors including the hexameric Origin Recognition Complex (ORC-1-6), and Cdc6 and Cdt1 proteins. Several mechanisms prevent Mcm helicase loading on chromatin outside the M/G1 phases to ensure that loading and activation of the Mcm helicase are temporally separated (Blow and Dutta 2005; Arias and Walter 2007).

During the second step, pre-RCs are converted into preinitiation complexes, in which activation of the Mcm helicase leads to DNA unwinding and initiation of DNA synthesis (“Origin firing”). This step is associated with the recruitment of many other factors to the origin by the S-phase promoting kinases CDK and Dbf4-dependent Cdc7 Kinase (DDK) (Labib 2010). These kinases promote the binding of Cdc45 and GINS (Go-Ichi-Ni-San in Japanese for 5-1-2-3 in reference to the four protein of the complex Sld5-Psf1-Psf2-Psf3) to Mcm2-7, resulting in the formation of the Cdc45-Mcm2-7-GINS (CMG) complex and in the helicase activation (Ilves *et al.* 2010). The mechanism of CMG assembly and activation is relatively well understood in budding yeast and has been reconstituted *in vitro* from purified components (Yeeles *et al.* 2015). Briefly, Cdk promotes CMG formation by phosphorylating Sld2 and Sld3 and thereby generates binding sites for the tandem BRCA1 C-terminus (BRCT) repeats in Dpb11/TopBP1/MUS-101 (Tanaka *et al.* 2007; Zegerman and Diffley 2007). Formation of the complex between Dbp11 and phospho-Sld2 is required for the recruitment of GINS and of the leading strand polymerase to replication origins (Labib 2010; Muramatsu *et al.* 2010). DDK phosphorylates Mcm2 and Mcm4 allowing the recruitment of Sld3 and in turn Cdc45 (Deegan *et al.* 2016).

Defects in DNA replication, for instance stalled replication forks, are sensed by the DNA replication checkpoint pathway, which prevents origin firing, stabilizes stalled replication forks, and facilitates the restart of collapsed forks (Harper and Elledge 2007; Cimprich and Cortez 2008). This pathway relies on the recruitment and activation of the PI3 kinase-related kinase Ataxia Telengectasia and Rad3 related (ATR) at sites of DNA damage. Once recruited, ATR phosphorylates and thereby activates the serine-threonine kinase Chk1 (Checkpoint kinase 1), which in turn blocks cell cycle progression (Guo *et al.* 2000; Liu *et al.* 2000). Several components play a dual role in DNA replication and DNA replication checkpoint signaling including TopBP1 and Claspin (Kumagai and Dunphy 2000; Burrows and Elledge 2008; Mordes *et al.* 2008).

At the end of DNA replication, when an ongoing DNA replication fork from one origin encounters an incoming DNA replication fork from an adjacent origin, DNA replication is stopped and the DNA replication fork helicase is disassembled (Bailey *et al.* 2015; Dewar *et al.* 2015). Recent work shows that in late S-phase in budding yeast, the AAA-ATPase Cdc48/p97 removes the replicative helicase from the chromatin after ubiquitination of Mcm7 by the Skp1-Cullin-F-box SCF^Dia2^ E3-Ligase (Maric *et al.* 2014). The role of Cdc48/p97 in CMG removal via Mcm7 ubiquitination is conserved in *Xenopus* (Moreno *et al.* 2014). However, the F-box protein Dia2 is not conserved, and the identity of the E3-enzyme involved in CMG removal in higher eukaryotes is still unknown (Ramadan *et al.* 2016).

SCF and related ubiquitin-ligases, generically termed Cullin-RING-E3-Ligases (CRLs), represent the most prominent family of E3-ubiquitin-ligases (Merlet *et al.* 2009; Lydeard *et al.* 2013). Proteomic analysis estimated that 20% of the proteome is regulated by CRL complexes (Soucy *et al.* 2009).

We are interested in studying the function and regulation of CRLs during cell cycle progression and development using the nematode *Caenorhabditis elegans*. We have identified the evolutionarily conserved CRL2^LRR-1^ E3-ligase as an important determinant of DNA replication integrity in this system (Merlet *et al.* 2010). Loss of LRR-1 function causes hyper-activation of the ATL-1 (Ataxia telangiectasia and Rad3 related protein-like)/DNA replication checkpoint pathway, both in the *C. elegans* germline and in the early embryos, resulting in sterility and embryonic lethality respectively (Merlet *et al.* 2010; Burger *et al.* 2013). In a screen for temperature-sensitive mutants that phenocopy *lrr-1(0)* null mutants (sterile mutants that are fertile in the absence of *atl-1*), we have identified a *cul-2* temperature-sensitive mutant (*cul-2(or209*ts*)*) clearly indicating that LRR-1 acts together with CUL-2 to maintain DNA replication integrity (Burger *et al.* 2013). The mode of action of the CRL2^LRR-1^ E3-ligase remains poorly understood.

Here, we report the identification of genes that, when reduced in function by RNAi, suppress partially *lrr-1(0)* and *cul-2(or209*ts*)* phenotypes. Through these screens we identified components and regulators of the CMG helicase, as well as all the core components of the DNA replication checkpoint pathway. These results indicate that CUL-2 and LRR-1 regulate the replisome to ensure DNA replication integrity in *C. elegans* and likely also in other organisms.

## Materials and Methods

### Nematode strains and culture conditions

*C. elegans* strains were cultured and maintained using standard procedures (Brenner 1974). Strains used in this study are listed in Table 1.

**Table 1:** Nematode strains used in this study

Strain	Genotype	Reference
WLP 144	*lrr-1(tm3543)II/mIn1[mIs14 dpy-10(e128)]II*; *ruls32[pie-1p*::*GFP*::*H2B + unc-119(+)]*	Merlet *et al.* 2010
WLP 267	*cul-2(or209*ts*)III*	Burger *et al.* 2013
TG1750	*gtIs61 [pie-1p*::*GFP(lap)*::*orc-2 + unc-119(+)]. ltIs37 [(pAA64) pie-1p*::*mCherry*::*his-58 + unc-119(+)] IV.*	Sonneville *et al.* 2012
TG1751	*gtIs62 [pie-1p*::*GFP(lap)*::*cdc-6 + unc-119(+)]. ltIs37 [(pAA64) pie-1p*::*mCherry*::*his-58 + unc-119(+)] IV.*	Sonneville *et al.* 2012
WLP 592	*leals31 [cdc-7*::*TY1*::*EGFP*::*3xFLAG + unc-119(+). ltIs37 [(pAA64) pie-1p*::*mCherry*::*his-58 + unc-119(+)] IV*	This study
RB1211	*C34G6.5(ok1267) I*	Caenorhabditis Genetics Center
WLP 223	*cul-2(or209*ts*)III*; *C34G6.5(ok1267) I*	This study

### RNAi suppressor screens

#### Visual lrr-1(0) suppressor screen:

The RNAi screen was performed in 24 well plates. We administered the dsRNA by the feeding method (Fraser *et al.* 2000; Kamath *et al.* 2001, 2003). We generated a subcollection of 150 RNAi clones targeting most of the genes involved in cell cycle control and DNA metabolism. All the RNAi clones, except the one targeting *clsp-1* (this study), were obtained from the commercially available RNAi clones from the MRC Geneservice (Cambridge, UK) (Supplemental Material, Table S1).

Briefly, 1 ml of RNAi cultures was grown overnight in 24-well plates in Lysogeny Broth media containing ampicillin (0.1 mg/ml) at 37°. Five hundred microliters of each culture were dispensed into the equivalent position of each of the 24-well plates containing Nematode Growth Media (NGM) with 1 mM Isopropyl β-D-1-thiogalactopyranoside (IPTG). Each plate contained a negative (*ctrl(RNAi)*) and a positive control (*atl-1(RNAi)*).

L1 larvae were prepared after treating *lrr-1(0)/mIn1* adult animals with axenizing solution (sodium hypochlorite and sodium hydroxide) and letting the embryos hatch in M9 solution. Around 50 larvae were then added to the 24-well (NGM) plates containing bacteria expressing dsRNA and incubated at 20° until the animals reached adulthood. The plates were then analyzed under a stereomicroscope equipped with fluorescence (Stereo Discovery V12 equipped with a Plan ApoS 1.5W FWD 30 mm Objective, Zeiss). The balancer chromosome *mIn1[dpy-10(e128) mIs14]* carries an integrated transgene (*mIs14*) such that heterozygous *lrr-1(0)/mIn1*, which express MYO-2::GFP in the pharynx, are easily distinguishable from the homozygous *lrr-1(0)* animals under the stereomicroscope. The morphology of the germline was visually evaluated in several *lrr-1(0)* homozygous mutant animals in each well. Wells containing *lrr-1(0)* homozygous with rescued germ cell proliferation defects were selected for further analysis and quantifications. The screen was performed in duplicate and the positive clones were retested in triplicate.

To quantify the suppression, *lrr-1(0)* animals were paralyzed in 20 mM levamisole, mounted on a 2% agarose pad and the fraction of animals with rescued germline was counted under a Zeiss microscope (AxioImager A1) equipped with fluorescence.

The DNA sequence of the RNAi bacterial clone present in the well was confirmed by sequencing.

#### cul-2(or209ts) suppressor screen:

The screen was performed in 24-well plates with each plate containing a negative (*ctrl(RNAi)*) and a positive control (*atl-1(RNAi*)). Twenty *cul-2(or209*ts*)* L1 larvae, prepared as described above, were inoculated in each well of the plate containing the RNAi bacterial clones and the plates were placed at permissive temperature (15°). When the animals reached the L4/young adult stage, the plates were transferred to semirestrictive temperature (23°) and the animals started to lay embryos. After 2−3 d, the level of suppression of the *cul-2(or209*ts*)* embryonic lethality was estimated by visual inspection of each well under a dissecting scope. The wells containing larvae, as opposed to dead embryos, were scored as positive.

#### cul-2(or209ts) suppression assays:

Feeding RNAi was performed in the same conditions as the screen. Five L1 larvae were fed until the L4/young adult stage at permissive temperature (15°) and then shifted at semirestrictive temperature (23°). After 11 hr, the adults were removed and the laid embryos were counted. The viability of the progeny was determined after incubating for another 24 hr at the same temperature. The percentage of viability was determined by dividing the number of hatched embryos by the total number of progeny and subtracting the value corresponding to the control (*cul-2(or209*ts*)* exposed to *ctrl(RNAi)*). The experiment was performed in triplicate.

### Time-lapse microscopy

For the visualization of early embryonic development in live specimens, embryos were obtained by cutting open gravid hermaphrodites using two 21-gauge needles. Embryos were handled individually and mounted on a coverslip in 7 μl of egg buffer (Shelton and Bowerman 1996). The coverslip was placed on a 2% agarose pad. Time-lapse Differential Interference Contrast (DIC) images were acquired by an Axiocam Hamamatsu ICcI camera (Hamamatsu Photonics, Bridgewater, NJ) mounted on a Zeiss AxioImager A1 microscope equipped with a Plan Neofluar 100×/1.3 NA objective (Carl Zeiss AG, Jena, Germany), and the acquisition system was controlled by Axiovision software (Carl Zeiss AG, Jena, Germany). Images were acquired at 10-sec intervals.

The timing of cytokinesis in P0 (measured at the time of cleavage furrow initiation) as well as that of Nuclear Envelope Breakdown in AB and P1 (measured at the time of nuclear membrane disappearance) was determined. The time separating cytokinesis in P0 from NEBD in either AB or P1 corresponds to interphase.

Live imaging was performed at 23° using a spinning disc confocal head (CSU-X1; Yokogawa Corporation of America) mounted on a Ti-E inverted microscope (Nikon) equipped with 491 nm and 561 nm lasers (Roper Scientific) and a charge-coupled device camera (Coolsnap HQ2; Photometrics). Acquisition parameters were controlled by MetaMorph software (Molecular Devices). In all cases a 60×, 1.4 NA PlanApochromat lens with 2 × 2 binning was used, and four z-sections were collected at 2-µm intervals every 25 sec. In Figure 5 and Figure 6, each image results from a single focal plane (performed with ImageJ/Fiji software).

### Plasmid construction

A modular polycistronic expression system was used to coexpress the four subunits of GINS complex in *Escherichia coli* (Tan 2001; Tan *et al.* 2005). To this end, Strep-SLD-5, 6x(His)-PSF-1, PSF-3, and PSF-2 were cloned on the polycistronic vector pST44 (Tan *et al.* 2005) generating the plasmid pLP963. The details of plasmid construction are available upon request.

### Biolistic transformation

The strain expressing GFP::CDC-7 was obtained by biolistic transformation of the fosmid 8859124759762056 E06 (Sarov *et al.* 2012). Biolistic transformation was performed as described previously (Praitis *et al.* 2001).

### Protein extracts and immunoprecipitation

For immunoprecipitation of SLD-5 fused to the Green Fluorescent Protein (GFP::SLD-5), total embryo extracts were prepared by cryolysis in lysis buffer (20 mM Hepes, pH 9, 150 mM NaCl, 2 mM MgCl_2_, 10 mM EDTA, 0.02% NPA, 1 mM DTT) supplemented with Complete protease inhibitor (Roche), and 800 Units of Universal Nuclease (Pierce). Extracts were incubated for 30 min at 4° under rotation and subsequently centrifuged for 30 min at 4°. The supernatant was then incubated with 20 µl of GFP-Trap beads (Rothbauer *et al.* 2008) or control beads for 2 hr at 4°. After three washes in ice-cold lysis buffer, proteins were eluted in 3xSDS Laemmli sample buffer after boiling for 10 min at 95° and subjected to immunoblot analysis.

For affinity purification of the *C. elegans* GINS complex, BL21 bacteria were transformed with the plasmid pLP963. The expression of the recombinant *C. elegans* GINS complex was induced by the addition of 1 mM IPTG to 1 l cultures of *E. coli* BL21 before incubation for 3 hr at 23°. After pelleting, the bacteria were resuspended in 20 ml 0.15 M NaCl, 0.5 mM dithiothreitol, 40 mM imidazole in 50 mM Hepes pH 6.8, before lysis by sonication. The soluble portion of the lysate was loaded on a 1 ml His-Trap HP Column (GE Healthcare). The column was washed with 10 volumes of lysis buffer, and bound proteins were eluted in lysis buffer containing 300 mM imidazole. Eluted proteins were then loaded on a 1 ml Strep-Trap Column (GE Healthcare). After washing steps, bound proteins were eluted in lysis buffer containing 2.5 mM desthiobiotin.

### Immunoblot analysis and antibodies

Standard procedures were used for SDS–PAGE and western blotting using Chemiluminescent HRP Substrate (Millipore). The following antibodies were used in this study: primary antibodies were directed against PSF-3 (rabbit, 1/1000) (Benkemoun *et al.* 2014), GFP (Rabbit, 1/500) (this study), Streptavidin coupled to horseradish peroxidase (Bertin Pharma) and 6xHis (Mouse, 1/1000) (Eurogentec). Secondary antibodies conjugated to peroxidase against rabbit or mouse were purchased from Sigma (1/3000).

### Statistical analysis

The results are presented as means ± SEM. In all graphs, data were compared by one sample or unpaired *t*-test. All calculations were performed with Prism software (Graphpad). * *P* < 0.05; ** *P* < 0.01; *** *P* < 0.001; ****P < 0.0001.

### Data availability

The authors state that all data necessary for confirming the conclusions presented in the article are represented fully within the article.

## Results and Discussion

### RNAi screens identified Cdc45-MCM-GINS (CMG) complex subunits and regulators as lrr-1(0) and cul-2(or209ts) suppressors

**Figure 1:**
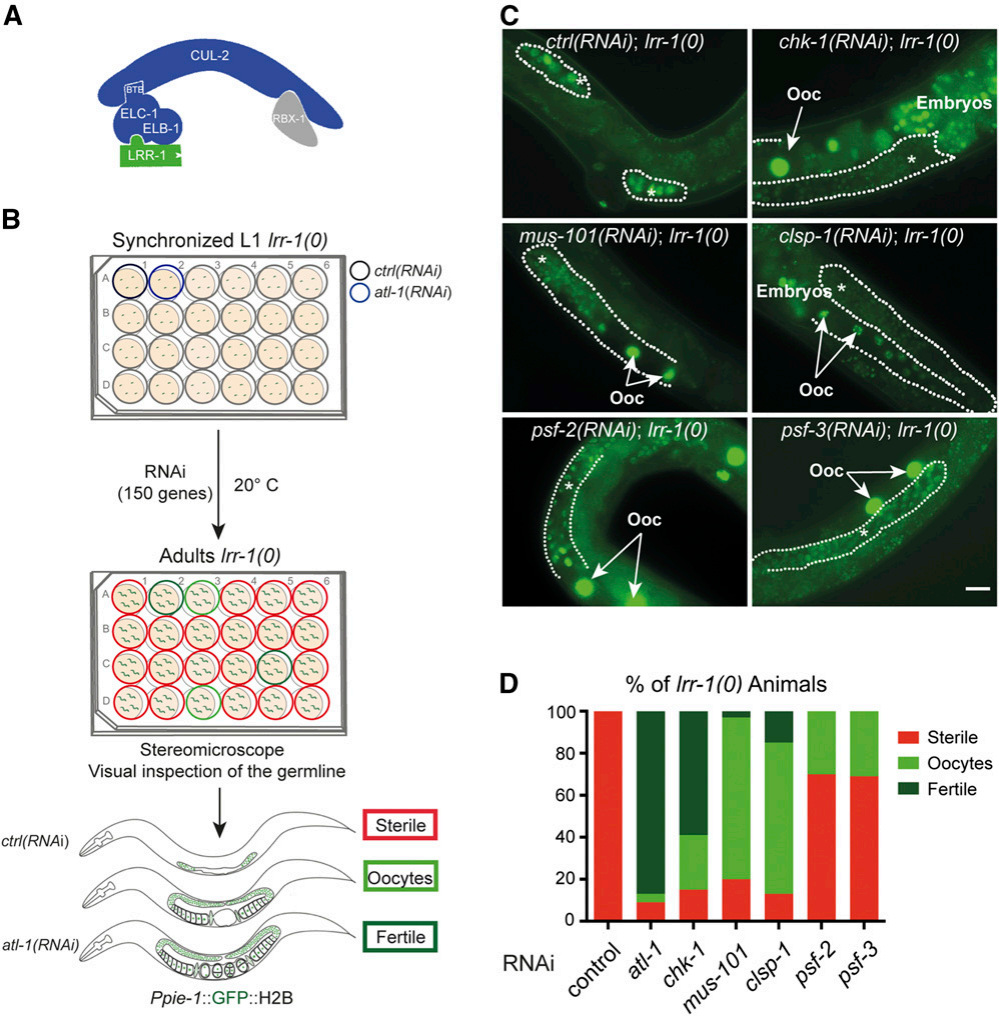
A visual RNAi-based screen for *lrr-1(0)* suppressors. (A) Schematic representation of the CRL2^LRR-1^ E3-Ligase. The evolutionarily conserved Leucine Rich Repeat protein LRR-1 (green) acts as a substrate-recognition subunit of Cullin-RING E3-ligase nucleated around CUL-2 (blue). ELC-1, in complex with ELB-1 (blue), serves as an adaptor and RBX-1 (gray) is the catalytic subunit. (B) Flow-chart of the visual RNAi-based screen for *lrr-1(0)* suppressors. Synchronized *lrr-1(0)/+* (not shown for simplicity) and *lrr-1(0)* L1 larvae expressing GFP::H2B in the germline were fed in several 24-well NGM plates until adulthood with bacteria expressing dsRNA targeting 150 genes involved in cell cycle control and DNA metabolism. Each plate contained a negative (*ctrl(RNAi)*) and a positive control (*atl-1(RNAi)*). The morphology of the germline of *lrr-1(0)* animals exposed to RNAi clones was visualized under a stereomicroscope equipped with fluorescence. The bottom panel shows a schematic representation of *lrr-1(0)* animals exposed to mock or *atl-1(RNAi)*. *lrr-1(0)* animals exposed to mock RNAi are fully sterile with a small germline (red rectangle) whereas *lrr-1(0)* animals exposed to *chk-1 or atl-1(RNAi)* produced gametes and embryos (dark green rectangle). In cases where the suppression is incomplete, the *lrr-1(0)* animals produce oocytes but no embryos (light green rectangle). (C) Representative fluorescence images of *lrr-1(0)* animals expressing the GFP::H2B transgene upon inactivation of the indicated genes. An asterisk and dotted lines indicate the position of the germ line. White arrows mark the position of the oocytes. Scale bar: 20 μm. (D) Graphs showing the percentage of *lrr-1(0)* animals, exposed to the indicated RNAi, presenting the different phenotypes: sterile animals with a small germline (red), animals producing oocytes (light green), fertile animals (dark green). More than 50 worms were analyzed in each condition.

To gain further insight into the role of the CRL2^LRR-1^ E3-ligase (Figure 1A), we conducted an RNA interference (RNAi)-based screen for suppressors of *lrr-1(0)* mutants. *lrr-1(0)* mutants are sterile with a small germline containing <50 germ cells (Merlet *et al.* 2010). We searched for genes that, when reduced in function by feeding RNAi, restored germ cell proliferation, the production of gametes and embryos to *lrr-1(0)* mutants. To facilitate the identification of *lrr-1(0)* suppressors using a stereomicroscope equipped with fluorescence, we used an *lrr-1(0)* strain expressing the histone H2B fused to the Green Fluorescent Protein (GFP::H2B) under the control of the germline specific promoter *pie-1*. As positive controls, we inactivated *chk-1* and *atl-1* in *lrr-1(0)* mutants as we have shown previously that inactivation of these genes restores the germ cell proliferation, germ line morphology, and fertility to *lrr-1(0)* mutants (Merlet *et al.* 2010).

We screened a subcollection of 150 genes encoding components involved in DNA metabolism and cell cycle control (Table S1). We inactivated these genes in *lrr-1(0)* L1 larvae by the feeding method and scored the morphology of the germline in adult animals (Figure 1B). Given that most of the selected genes in our subcollection are essential genes, we performed the screen in conditions allowing the partial inactivation of the target genes by RNAi (*Materials and Methods*).

As shown in Figure 1C, *lrr-1(0)* mutants are sterile with a small germline whereas *chk-1(RNAi)*; *lrr-1(0)* mutants produce gametes and embryos, readily observable through the expression of GFP::H2B that accumulates in the nuclei of oocytes and embryos (Merlet *et al.* 2010). Besides *atl-1* and *chk-1*, the positive controls, we identified in this screen four additional genes: *mus-101*/TopBP1, F25H5.5/*clsp-1*, *psf-2*, and *psf-3*, which regulate DNA replication and the DNA replication checkpoint pathway.

Reduction in function of these genes partially restored germ cell proliferation and the production of oocytes in *lrr-1(0)* mutants, with their nuclei readily detectable as bright green spots under the stereomicroscope (Figure 1C arrows). Quantification revealed that 100% of *lrr-1(0)* mutants produced sterile animals containing only a few germ cells whereas inactivation of *psf-2* and *psf-3* restored germ cell proliferation and oocyte differentiation in about 30% of animals (*n* > 50) (Figure 1D). Suppression of the *lrr-1(0)* phenotype was more pronounced upon inactivation of *clsp-1* or *mus-101* with >80% of *lrr-1(0)* animals producing oocytes (*n* > 50) (Figure 1D). These results indicate that down-regulating *psf-2*, *psf-3*, *mus-101*, and *clsp-1* partially suppresses the germ cell proliferation defects of homozygous *lrr-1(0)* null mutant animals.

In parallel and complementary to this visual screen, we conducted an RNAi screen for suppressors of the embryonic lethality of the *cul-2(or209)* temperature-sensitive mutant using the same subcollection of genes that was used for the *lrr-1(0)* suppressor screen. We screened for genes that, when reduced in function by feeding RNAi, allow embryos from homozygous *cul-2(or209*ts*)* mutants to hatch at a semirestrictive temperature (23°).

**Figure 2:**
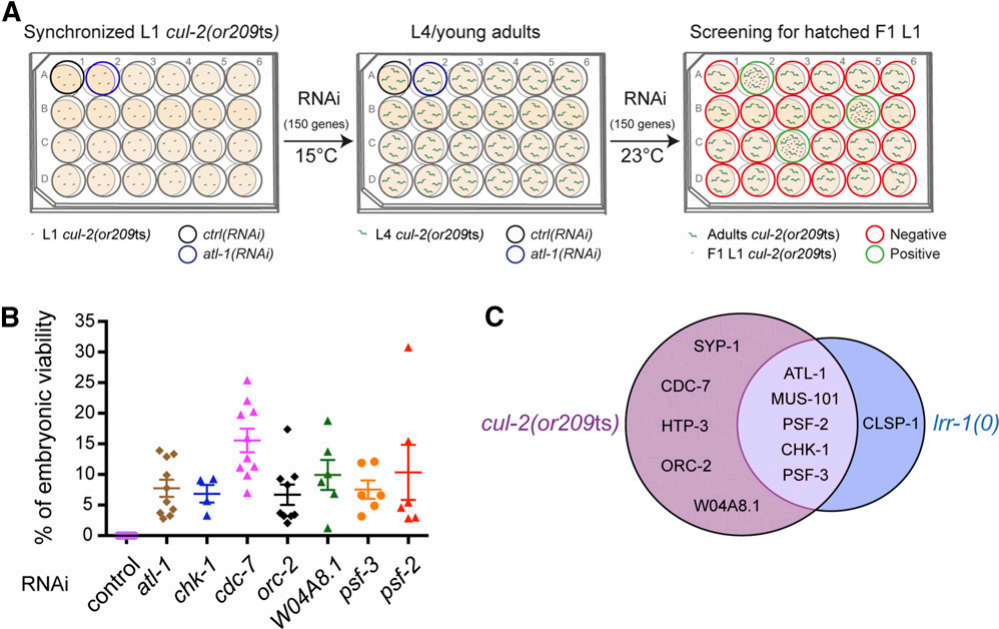
An RNAi screen for suppressors of the embryonic lethality of the *cul-2(or209*ts*)* temperature-sensitive allele. (A) Schematic representation of the *cul-2(or209*ts*)* RNAi-based suppressor screen. Synchronized *cul-2(or209*ts*)* L1 larvae were fed in 24-well plates with bacteria expressing dsRNA at 15° until the L4/young adult stage, then the plates were placed at the semipermissive temperature (23°). After 2 d, the wells containing larvae, as opposed to dead embryos, were selected as positive (green wells). The black well contained a negative (*ctrl*) and the blue well a positive (*atl-1*) control. (B) Graphs showing the percentage of viability of *cul-2(or209*ts*)* animals after RNAi-mediated depletion of the indicated genes. Each dot on the graph corresponds to an independent experiment. Numerical values and statistical analysis are provided in Table S2. (C) Genes identified in the *cul-2(or209*ts*)* (purple circle), *lrr-1(0)* suppressor screens (blue circle), and in both screens.

L1 larvae from *cul-2(or209*ts*)* mutants were grown at a permissive temperature (15°) in NGM RNAi plates until the L4 stage and then shifted to 23° (Figure 2A). *cul-2* mutants exposed to control RNAi produced nonviable embryos whereas reducing the function of *atl-1*, *mus-101*, *htp-3*, *chk-1*, and *syp-1* (Burger *et al.* 2013) but also of *orc-2*, *psf-2*, *psf-3*, *cdc-7*, and W04A8.1, significantly and reproducibly restored viability of *cul-2(or209*ts*)* embryos.

To quantify the level of suppression, we performed the experiments in the same conditions as the screen but after shifting the *cul-2(or209*ts*)* animals for 11 hr at 23°, adults were removed and laid embryos were counted. After 2 d, the percentage of embryos that were able to hatch was determined. We repeated the experiments at least five times for each gene and reproducibly found that partial inactivation of these genes restored viability to the *cul-2(or209*ts*)* mutant embryos (Figure 2B).

**Figure 3:**
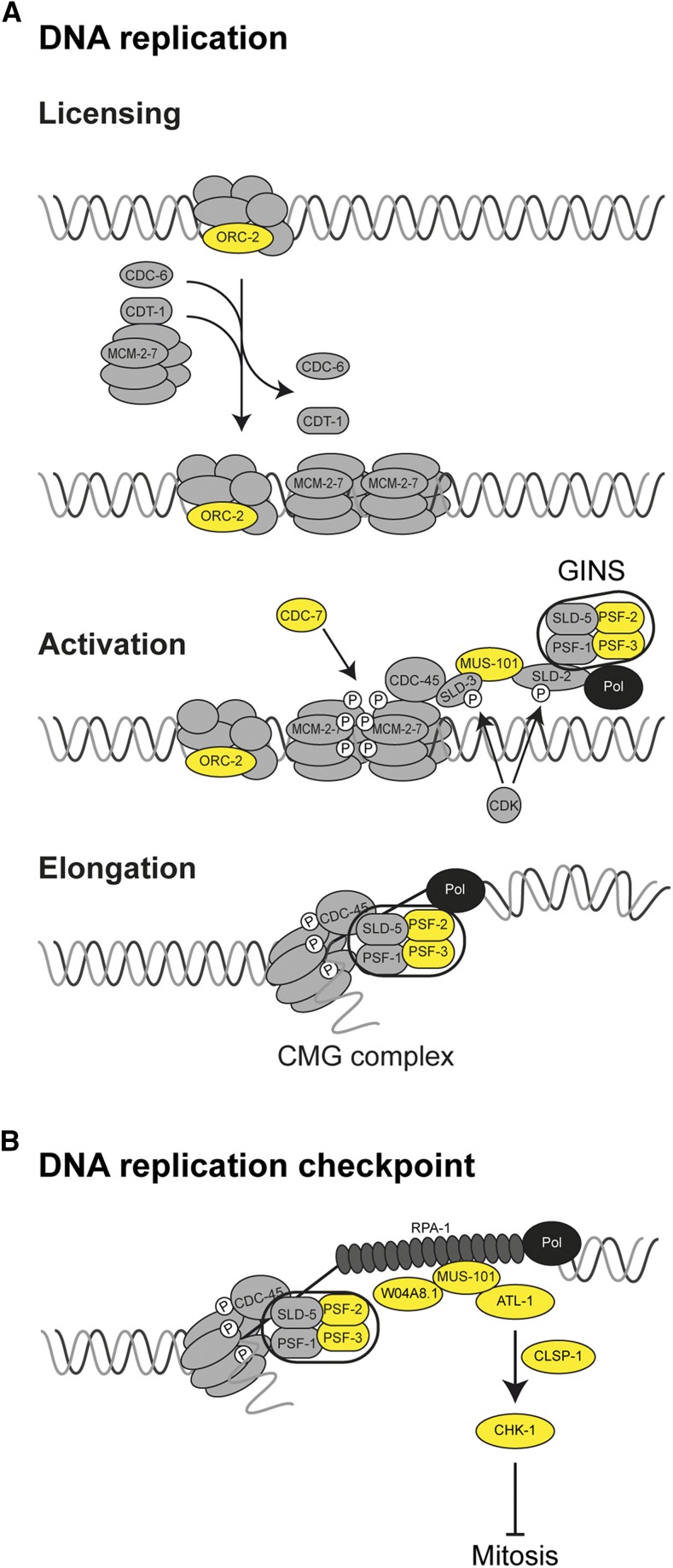
The CRL2 ^LRR-1^ E3-ligase controls DNA replication integrity. Schematic representation of (A) the DNA replication and (B) the DNA replication checkpoint pathway in eukaryotes. DNA replication occurs in two steps that are regulated by CDK and DDK activities. Factors identified in the *lrr-1(0)* and *cul-2(or209*ts*)* suppressor screens are highlighted in yellow.

Overall, these two screens led to the identification of 11 suppressors that fall in two main categories: meiosis (SYP-1, HTP-3), DNA replication and DNA replication checkpoint pathway activation (ORC-2, CDC-7, PSF-2, PSF-3, ATL-1, CHK-1, MUS-101, CLSP-1, W04A8.1). Most of these genes were identified in both screens supporting an essential role of the CRL2^LRR-1^ E3-Ligase in DNA replication integrity (Figure 2C and Figure 3).

### Function of cul-2 and lrr-1 mutant suppressors

#### Meiosis:

HTP-3 and SYP-1 are required during meiosis to promote the formation of the synaptonemal complex (SC) between homologous chromosomes (MacQueen *et al.* 2002, 2005; Goodyer *et al.* 2008). HTP-3 acts upstream of SYP-1 in the assembly of the SC. We showed that a partial *htp-3* loss-of-function allele (*htp-3(vc75)*) suppresses partially *lrr-1(0)* mutant sterility, therefore providing evidence that one function of LRR-1 is to prevent the premature assembly of the SC in the mitotic region of the germline (Burger *et al.* 2013).

#### DNA replication:

ORC-2 is part of the Origin Recognition Complex (ORC) that together with Cdt1 and Cdc6 loads the Mcm2-7 complex onto chromatin. It is believed that ORC binds first and then recruits Cdc6 and Cdt1 (Blow and Dutta 2005). In early *C. elegans* embryos, where the cell cycle alternates between the S and M phase without intervening gap phases, GFP::ORC-2 is exclusively enriched on the chromatin in mitosis similarly to the other licensing factors CDC-6 and CDT-1 (Sonneville *et al.* 2012). These factors, particularly ORC-2 and CDC-6, are excluded from interphase nuclei as a result of active nuclear export that depends on the exportin Crm1/XPO-1. The active nuclear export of these licensing factors is required to prevent rereplication in early *C. elegans* embryos (Sonneville *et al.* 2012). Similar types of regulation take place in other organisms to inactivate licensing factors and to prevent DNA rereplication (Blow and Dutta 2005; Arias and Walter 2007).

PSF-2 and PSF-3 are two subunits of the GINS complex that also contains the SLD-5 and PSF-1 subunits. *psf-1* was also present in our subcollection of targeted genes; however, it was not recovered in this screen, possibly because its inactivation was either too severe or inefficient by RNAi.

**Figure 4:**
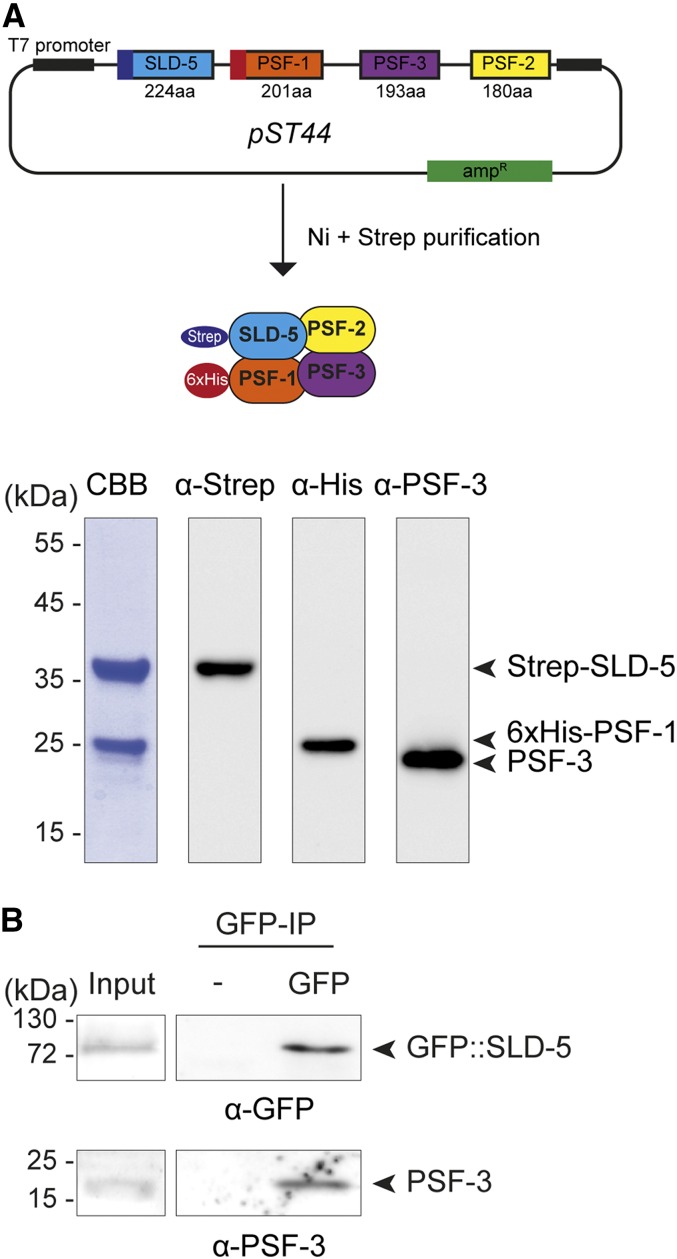
Characterization of the *C. elegans* GINS complex. (A) Schematic representation of the plasmid expressing the four subunits of the GINS complex: SLD-5, PSF-1, PSF-2, and PSF-3. SLD-5 and PSF-1 were tagged with the Strep and 6xHis tags respectively (upper panel). Coomassie blue (CBB) staining and immunoblot analysis of the double purification of the *C. elegans* GINS complex from *E. coli* using antibodies against Strep, 6xHis, and PSF-3 (lower panel). (B) GFP immunoprecipitation using GFP-TRAP or control (–) beads from early embryos expressing GFP::SLD5; mCherry::H2B. Immunoprecipitates were subjected to immunoblot analysis using GFP and PSF-3 antibodies.

To test whether the *C. elegans* GINS subunits assemble as a complex, we expressed the four subunits from a polycistronic vector in *E. coli* and purified the complex using double tag affinity purification (see *Materials and Methods*). Through this approach, we purified the GINS complex and confirmed the presence of PSF-1, PSF-3, and SLD-5 suggesting that the four *C. elegans* GINS subunits can readily assemble as a stable complex (Figure 4A). Consistently, we found that PSF-3 coimmunoprecipitates with GFP::SLD-5 from *C. elegans* embryo extracts (Figure 4B). PSF-3 localizes into the nucleus specifically in interphase in early embryos (Benkemoun *et al.* 2014), which is consistent with its role in DNA replication. These results strongly suggest that similarly to other organisms, *C. elegans* PSF-2, PSF-3, SLD-5, and PSF-1 form a stable complex that is required for DNA replication. Accordingly, inactivation of the four GINS subunits delays the division of the P1 blastomere at the two-cell stage (Benkemoun *et al.* 2014), presumably as a result of a defect in DNA replication, eventually leading to the activation of the DNA replication checkpoint (Encalada *et al.* 2000; Brauchle *et al.* 2003).

MUS-101 is the *C. elegans* homolog of the BRCA1 C-Terminus **(**BRCT) repeats containing protein Dbp11/TopBP1 that plays a dual role in CMG complex assembly and DNA replication checkpoint activation (Garcia *et al.* 2005; Wardlaw *et al.* 2014). BRCT domains mediate protein–protein and protein–DNA interactions and are heavily represented among proteins involved in the DNA damage response (Gerloff *et al.* 2012). In particular, the BRCT domain is a phospho-binding domain (Yu *et al.* 2003), which allows dynamic interaction with partners through the activity of various kinases and phosphatases. Although the TopBP1 orthologs each function in replication initiation and checkpoint activation, their modular compositions are different (Wardlaw *et al.* 2014). For instance, budding yeast Dpb11 and fission yeast Rad4 contain four BRCT repeats arranged as two pairs whereas *C. elegans* MUS-101 contains six BRCT repeats.

In *C. elegans*, MUS-101 is essential for DNA replication (Holway *et al.* 2005). Similarly to its yeast counterpart, MUS-101 interacts with SLD-2 phosphorylated by the Cdk1 kinase (Gaggioli *et al.* 2014). However, whereas Sld2 interacts with Dbp11-BRCT repeats 3/4 in yeast, SLD-2 interacts with MUS-101-BRCT repeats 5/6 in *C. elegans* (Gaggioli *et al.* 2014). Consistent with its role in DNA replication, MUS-101 localizes to the nucleus in interphase in early *C. elegans* embryos (Benkemoun *et al.* 2014).

CDC-7 forms a complex with Dbf4 generically called DDK (Dbf4-Dependent Kinase). DDK is essential for the activation of the CMG helicase by phosphorylating several MCM subunits (Labib 2010; Deegan *et al.* 2016).

**Figure 5:**
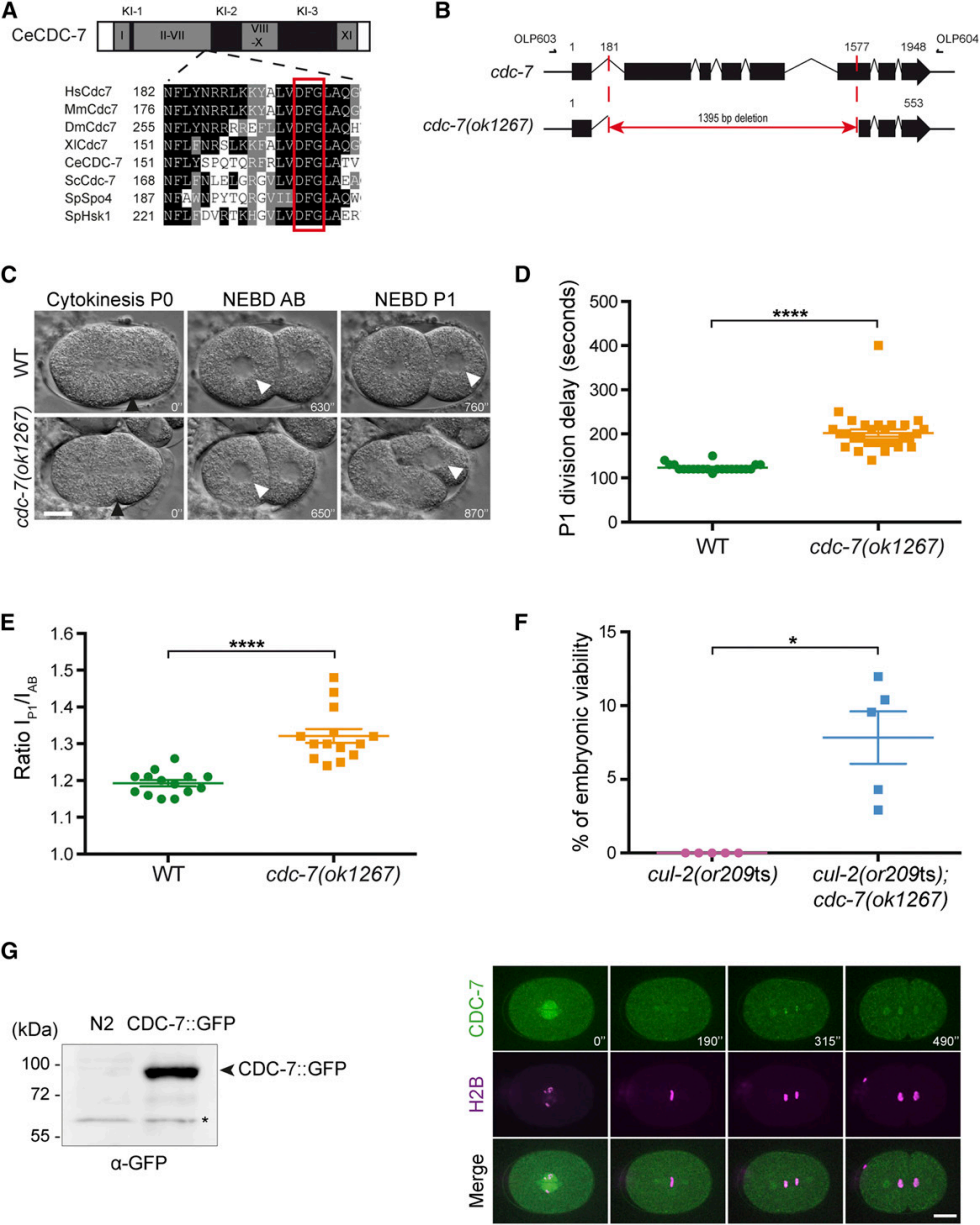
*cdc-7* characterization in *C. elegans*. (A) Domain organization of *C. elegans* CDC-7. CDC-7 contains the typical kinase domains (I-XI) and the specific kinase inserts (KI) shown in gray and black respectively. Multiple protein sequence alignments corresponding to the region containing the conserved DFG motif in the II-VII Kinase domain in different organisms is presented. (B) Schematic representation of the *cdc-7(ok1267)* deletion allele. Deletion removes 1395 base pairs between the middle of intron 1 and the middle of exon 6. The position of the oligonucleotides (OLP603: 5′ agaaggacaactggctccaa 3′ and OLP604: 5′ caacacagcaagcgagaaaa 3′) used to genotype the wild type and *cdc-7(ok1267)* deletion strains are indicated. (C) DIC images from time-lapse video recording wild-type (N2) and *cdc-7(ok1267)* embryos. Black arrowheads indicate cleavage furrow ingression; white arrowheads indicate nuclei undergoing NEBD, which is apparent by loss of the smooth line corresponding to the nuclear envelope. Cleavage furrow ingression at the onset of cytokinesis in P0 is defined as *t* = 0, and the time after that is indicated in seconds. Scale bar: 10 μm. (D) Graph reporting the elapsed time ± SEM between AB and P1 cytokinesis (in seconds) in wild-type (N2) (*n* = 22) and in *cdc-7(ok1267)* mutants (*n* = 30). (E) Average ratios ± SEM of the duration of interphase in P1 over the duration of interphase in AB [(I_P1_)/(I_AB_)] (RI) in embryos of the indicated genotypes (*n* = 14). The asterisks indicate that the difference with wild type is statistically significant. See Table S2 for numerical values and statistical analysis. (F) Double *cul-2(or209*ts*)*; *cdc-7(ok1267)* mutant analysis. The percentage of embryonic viability of the double *cul-2(or209*ts*)*; *cdc-7(ok1267)* mutant was determined in the condition of the *cul-2(or209*ts*)* suppressor screen and plotted. The experiment was repeated five times. Asterisk indicates a significant difference (*P* = 0.011). (G) Western blot analysis of total embryo extracts expressing GFP::CDC-7 compared to the wild type (N2) using an anti-GFP antibody. Arrow indicates the GFP::CDC-7. Asterisk indicates an unspecific band (left panel). Time-lapse spinning disk confocal micrographs of an early embryo expressing GFP::CDC-7 and mCherry::H2B. Scale bar: 10 μm (right panel).

A Cdc7 regulating subunit, which may potentially bear similarity to Dbf4, remains to be discovered in *C. elegans*. In contrast to Dbf4, Cdc7 is relatively well conserved in *C. elegans*. Multiple protein sequence alignments confirmed the presence of characteristic features in CDC-7 including large inserts between the conserved kinase catalytic subdomains and the conserved DFG motif that is critical for phosphorylation (Endicott *et al.* 2012) (Figure 5A and Figure S1).

Whereas *cdc-7* is conserved in *C. elegans*, it has not yet been characterized. A deletion allele (*cdc-7(ok1267)*) that removes most of the *cdc-7* gene has been obtained by reverse genetics. PCR amplification and DNA sequencing confirmed the presence of a deletion of 1395 bp in the *cdc-7(ok1267)* mutant animals. This large deletion removes most of the kinase subdomains, including the DFG motif, most likely generating an inactive CDC-7 Kinase (Figure 5B). The strain harboring this allele is viable suggesting that CDC-7 function might not be essential in *C. elegans*. However, *cdc-7(ok1267)* mutants grow slowly as compared to wild type and *cdc-7(ok1267)* embryos present a slight delay in the division of the P1 blastomere, resulting in a significant increase in the asynchrony of cell division between AB and P1 blastomeres (Figure 5, C and D). This increase in cell asynchrony is not merely resulting from a slower cell cycle in AB and P1 but to a specific delay in the division of the P1 blastomere, as revealed by an increase in the ratio of the duration of interphase in P1 over the duration of interphase in AB (Figure 5, C and E). This defect is a characteristic feature of a slower DNA replication.

Combining the *cdc-7* deletion allele with the *cul-2(or209*ts*)* allele significantly restored viability of the *cul-2(or209*ts*)* strain, consistent with the results obtained by feeding RNAi (Figure 5F and Figure 2B).

To further characterize CDC-7 in *C. elegans*, we generated a strain expressing GFP::CDC-7 (Figure 5G, left panel) and used spinning disk confocal microscopy to monitor its subcellular localization in early embryos. GFP::CDC-7 localized to the nucleus in interphase, and to the centrosomes and the mitotic spindle. During metaphase, GFP::CDC-7 was excluded from the chromatin but started to accumulate on the chromatin in anaphase (Figure 5G, right panel). This localization is consistent with a role of CDC-7 in DNA replication.

It is, however, surprising that *cdc-7* might not be an essential gene in *C. elegans* given it is essential in most systems studied so far. Several mutations, bypassing the need of Cdc7 or Dbf4, have led to identification of *mcm* mutation in budding yeast, raising the attractive possibility that these *mcm* mutations might cause structural changes in the Mcm2-7 complex, possibly mimicking what normally happens when Mcm2-7 subunits are phosphorylated by Cdc7-Dbf4 (Labib 2010). It is conceivable that MCM subunits in *C. elegans* already adopt a structure that yeast MCM subunits would only adopt upon phosphorylation by Cdc7. Alternatively, Cdk1 or potentially other kinase(s) might act redundantly with CDC-7 in worms. All together, these results indicate that CDC-7 contributes to DNA replication efficiency, and that its inactivation by RNAi, or by using a genetic mutant, partially suppresses *cul-2(or209*ts*)* embryonic lethality.

#### DNA replication checkpoint:

Our screens led to the identification of most of the worm homologs of the DNA replication checkpoint pathway components including MUS-101/TopBP1, ATL-1/ATR, MCPH-1/Microcephalin, CLSP-1/Claspin, and CHK-1/Chk1.

ATL-1 and CHK-1 are the *C. elegans* homologs of ATR and CHK1, respectively (Brauchle *et al.* 2003; Garcia-Muse and Boulton 2005). Inactivation of *atl-1* or *chk-1* abrogates the DNA replication checkpoint response in *C. elegans* and robustly suppresses *lrr-1(0)* and *cul-2(or209*ts*)* mutant phenotypes (Merlet *et al.* 2010; Burger *et al.* 2013). Little is known, however, about the activation mechanism of ATL-1 in *C. elegans*. By contrast, the mechanism of ATR activation has been extensively studied in *Xenopus* or in human cells (Cimprich and Cortez 2008; Nam and Cortez 2011). ATR activation occurs after the following steps: first ATR is recruited to ssDNA via its partner ATR interacting protein (ATRIP), which binds to ssDNA-RPA1 complexes (Cortez *et al.* 2001; Zou and Elledge 2003). Second, the 9-1-1 complex composed of Rad9-Rad1-Hus1 is loaded onto DNA damaged sites by the clamp loader Rad17 (Delacroix *et al.* 2007; Lee *et al.* 2007). Third, ATR autophosphorylation promotes its binding to TopBP1 (Liu *et al.* 2011) and in turn TopBP1 stimulates ATR kinase activity toward its substrates (Kumagai *et al.* 2006; Mordes *et al.* 2008). Recent work challenged this model and showed that TopBP1 directly interacts with RPA-coated ssDNA via its BRCT2 domain and acts upstream of the 9-1-1 complex (Yan and Michael 2009; Acevedo *et al.* 2016).

##### MUS-101:

The mechanism of MUS-101 recruitment to the sites of stalled replication forks or DNA damage remains to be investigated in *C. elegans*. It is worth mentioning that an ATRIP homolog is not apparent in *C. elegans* and although *hpr-17* (homologous to human *rad17*), *hpr-9* (human *rad9*), *mrt-2* *hus-1* were present in our subcollection of targeted genes, none of them were identified in our screens. Although we cannot exclude the possibility that RNAi against these genes was inefficient in our working conditions, it is possible that MUS-101 is directly recruited to ssDNA/RPA-1 complexes in *C. elegans*, similarly to the situation in *Xenopus* (Acevedo *et al.* 2016).

In *Xenopus*, the roles of TopBP1 in DNA replication initiation and ATR signaling are distinct (Kumagai *et al.* 2006; Yan *et al.* 2006). *mus-101* depletion by feeding RNAi mimics a hypomorphic condition and confers sensitivity to the DNA damaging agent methyl methanesulfonate (MMS), but does not block DNA replication (Holway *et al.* 2005). These results suggest that *mus-101* depletion by feeding RNAi may suppress *cul-2(or209*ts*)* and *lrr-1(0)* mutant phenotypes by altering the DNA replication checkpoint, but it is also possible that a role in DNA replication is responsible. Accordingly, a slight increase in the AB-P1 asynchrony, which is suggestive of mild DNA replication defects, has been reported in *mus-101(RNAi)* embryos obtained by feeding RNAi (Benkemoun *et al.* 2014).

##### W04A8.1:

The protein MCPH1/BRIT, which is mutated in microcephaly (Jackson *et al.* 2002), is also an earlier responder in the DNA damage response and regulates the recruitment of several downstream factors. In particular, MCPH1 interacts with TopBP1 and facilitates its recruitment to the sites of DNA lesions (Zhang *et al.* 2014). W04A8.1, which has not yet been characterized in worms, encodes a protein weakly similar to human MCPH1. While their primary sequence similarity is very subtle (only being detectable with psi-BLAST on NCBI-nr, using *C. briggsae* CBG13622 as a query sequence), both W04A8.1 and microcephalin have similar ordering of BRCT domains (one N-terminal, two C-terminal). Based on the recent results showing a role of microcephalin in TopBP1 recruitment at the sites of DNA damage (Zhang *et al.* 2014), it is tempting to speculate that W04A8.1/MCPH-1 fulfils a similar role in *C. elegans*.

##### CLSP-1:

Claspin acts as a mediator protein in checkpoint regulation by facilitating the phosphorylation of Chk1 by ATR and stimulating the autophosphorylation of Chk1 (Kumagai and Dunphy 2000; Kumagai *et al.* 2004). In turn, Chk1 blocks cell cycle progression by acting on the Cdc25 phosphatase (Guo *et al.* 2000; Liu *et al.* 2000). In early *C. elegans* embryos, CLSP-1 localizes to the nucleus in interphase (Dorfman *et al.* 2009). Although CLSP-1 has not yet been linked to the DNA replication checkpoint pathway in *C. elegans*, our finding that its inactivation suppresses *lrr-1(0)* mutant phenotype strongly suggests that is also required for activation of the DNA replication checkpoint in *C. elegans*.

Overall these two complementary screens led to the identification of 11 genes. As discussed earlier, feeding RNAi provides a very unique window of protein depletion that often mimics hypomorphic conditions, as described for the *mus-101* gene (Holway *et al.* 2005). Essential genes such as *psf-2*, *psf-3*, or *mus-101* would have been potentially difficult to identify if screening a mutant collection for suppression. Noteworthy, we originally identified the licensing factors *cdt-1*, *cdc-6*, and *mcm-6* as *cul-2(or209*ts*)* suppressors but the suppression was not reproducible, possibly because depletion of these essential genes by feeding RNAi was too severe in our conditions. Conversely, some other genes might have been missed because their depletion by feeding RNAi was inefficient. The main issue with feeding RNAi is that the level of gene depletion can be variable from one to another experiment. Furthermore, the developmental stage at which we shift the *cul-2(or209*ts*)* animals to 23° adds some variability to the experiment as this mutation affects CUL-2 production at high temperature (Burger *et al.* 2013). We circumvented this problem by multiplying the experiments and performing a quantitative analysis of the suppression.

Moreover, we have confirmed that some genetic mutants of identified suppressors, in particular *cdc-7(ok1267)* (this study), *atl-1(0)*, and *htp-3(vc75)* (Merlet *et al.* 2010; Burger *et al.* 2013), also suppress the *lrr-1* or *cul-2* mutant phenotypes. Remarkably, we have identified the DNA replication checkpoint pathway including the known checkpoint factors ATL-1, CHK-1, and MUS-101. In addition, we have identified the potential *C. elegans* homologs of claspin but also microcephalin, which is emerging as a central component of the checkpoint pathway, required for ATR signaling amplification (Zhang *et al.* 2014).

These screens clearly point out a role of the CRL2^LRR-1^ E3-Ligase in DNA replication and/or DNA replication checkpoint regulation. What could be the role of the CRL2^LRR-1^ E3-Ligase in these processes? For instance, DNA replication checkpoint components might be substrate(s) of the CRL2^LRR-1^ E3-Ligase, such that in the absence of the E3-ligase, these factors would accumulate causing a constitutive activation of the DNA replication checkpoint. Although we did not detect any changes in protein levels for the checkpoint factors CHK-1, MUS-101, and CSLP-1 upon inactivation of *cul-2* or *lrr-1* (data not shown), we cannot exclude this possibility.

**Figure 6:**
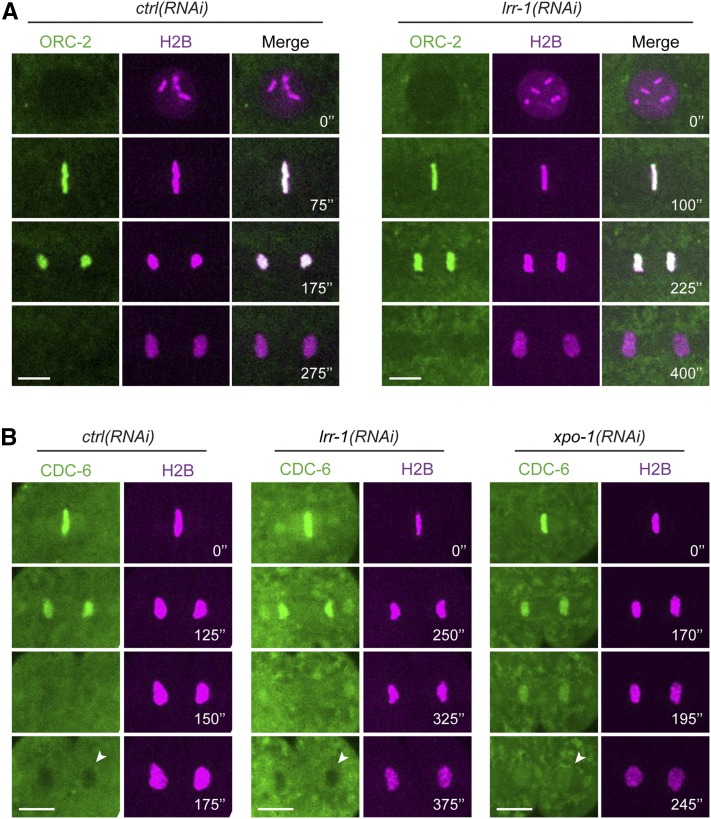
ORC-2 and CDC-6 localization is unaffected in *lrr-1(RNAi)* embryos. Time-lapse spinning disk confocal images of (A) *ctrl(RNAi)* and *lrr-1(RNAi)* dividing embryos in P1 blastomere expressing GFP::ORC-2 and mCherry::H2B and of (B) *ctrl(RNAi)*, *lrr-1(RNAi)*, and *xpo-1(RNAi)* anaphase embryos in P1 blastomere expressing GFP::CDC-6 and mCherry::H2B. CDC-6 is excluded from the telophase nuclei in *ctrl(RNAi)* and *lrr-1(RNAi)* embryos, in contrast to *xpo-1(RNAi)* embryos, as indicated by the arrowheads. Time interval is indicated in seconds. Scale bar: 5 μm.

Alternatively, the DNA replication checkpoint might be activated as a consequence of DNA replication defects. Consistent with this hypothesis, we previously found that ssDNA/RPA-1 foci accumulated in *lrr-1(0)* mutant germ cells. Furthermore, we noticed that a fraction of *atl-1(RNAi)*; *lrr-1(0)* germ cells accumulated with a DNA content greater than 4N, which was suggestive of DNA rereplication (Merlet *et al.* 2010). Several mechanisms preventing DNA rereplication after S-phase through inactivation of pre-RC components have been described in *C. elegans*. CDT-1 is targeted for degradation by the CRL4^Cdt2^ E3-Ligase (Zhong *et al.* 2003), whereas CDC-6 and ORC-2 are actively exported to the cytoplasm by the XPO-1 Exportin (Sonneville *et al.* 2012). To investigate whether LRR-1 regulates the pre-RC, we examined the localization of the pre-RC components CDT-1, CDC-6, and ORC-2 in *lrr-1(RNAi)* embryos. Indirect immunofluorescence revealed that CDT-1 levels and localization were not affected in *lrr-1(RNAi)* embryos (data not shown). Likewise, spinning confocal microscopy revealed that GFP::CDC-6 and GFP::ORC-2 were normally exported to the cytoplasm in *lrr-1(RNAi)*, in contrast to *xpo-1(RNAi)* embryos (Figure 6). Taken together, these results indicate that LRR-1 does not control the stability or the localization of the licensing factors CDT-1, CDC-6, and ORC-2, at least in early embryos.

Finally, we also tested whether CMG components, in particular PSF-2 and PSF-3, were misregulated in *lrr-1(0)* RNAi embryos. In *C. elegans*, inactivation of the CDC-48 segregase, and its cofactors NPL-4 and UFD-1, stabilizes CDC-45 and GINS on chromatin up to mitosis (Mouysset *et al.* 2008; Franz *et al.* 2011). We thus tested whether PSF-3 similarly accumulated on chromatin in mitotic *atl-1(RNAi)*; *lrr-1(0)* embryos. However, we did not detect any stabilization of PSF-3 on the mitotic chromatin in these embryos (data not shown).

Importantly, in a screen for E3-Ligases driving CMG disassembly in *C. elegans*, R. Sonneville and K. Labib have recently discovered that the CRL2^LRR-1^ E3-ligase promotes CMG disassembly in S-phase through MCM-7 ubiquitination. However, a second pathway contributes to CMG disassembly in mitosis (R.S and K.L, personal communication). Given that the second pathway remains active in absence of *lrr-1*, this explains why we did not detect any PSF-3 stabilization on the mitotic chromatin in *atl-1(RNAi)*; *lrr-1(0)* embryos. The persistence of CMG components on chromatin in *lrr-1(RNAi)* and *cul-2(RNAi)* is transient and only occurs during prophase (R.S. and K.L., personal communication).

Whether CMG persistence on the chromatin in prophase causes activation of the DNA replication checkpoint in *lrr-1* and *cul-2* mutants remains to be investigated. Based on our genetic results, it would be tempting to speculate that persistence of the CMG on chromatin is leading to DNA replication termination defects, resulting in the activation of the DNA replication checkpoint. In that scenario, reducing CMG levels, or assembly, by down-regulating *orc-2*, *psf-2*, *psf-3*, *cdc-7*, or *mus-101* would suppress *lrr-1(0)* and *cul-2(or209*ts*)* mutant phenotypes. A reduced number of loaded and activated CMG on the chromatin would compensate for a defect in CMG unloading.

Consistent with this possibility, a recent study reported that inhibition of CMG unloading prevents completion of DNA synthesis in *Xenopus* extracts leading to the accumulation of ssDNA (Bailey *et al.* 2015). However another study, analyzing the behavior of two converging forks replicating a plasmid in *Xenopus* egg extracts, showed that CMG removal is only triggered once DNA replication is completed (Dewar *et al.* 2015). They provided compelling evidence that when forks converge, the CMG passes over the ssDNA–dsDNA junction and keeps moving along the dsDNA such that the final ligation steps occurs prior to CMG dissociation (Dewar *et al.* 2015). In this context, CMG persistence on chromatin does not cause accumulation of ssDNA. However, the authors analyzed only two converging forks on a plasmid, while the first study looked at replication termination of the entire genome, which is known to occur asynchronously. It thus remains entirely possible that persisting CMG complexes travel on the chromatin and interfere with parts of the genome that are not yet fully replicated, thereby generating DNA replication defects eventually leading to the accumulation of ssDNA. Given that a stretch of 200–1000 single-stranded nucleotides is sufficient to trigger a robust checkpoint response *in vitro* (Choi *et al.* 2010), it is possible that CMG temporarily persisting on the chromatin in *lrr-1(RNAi)* embryos leads to DNA replication termination defects and DNA replication checkpoint activation. Further work will be required to test the functional consequences of CMG accumulation on the chromatin. In particular, it will be critical to test whether nonubiquitinated forms of Mcm7, which are expected to cause CMG persistence on the chromatin, activate the DNA replication checkpoint. It remains however entirely possible that LRR-1 promotes ubiquitination of other DNA replication factor(s) whose accumulation in *lrr-1(0)* and *cul-2* mutants causes activation of the DNA replication checkpoint. Identifying the CRL2^LRR-1^ substrates regulating DNA replication remains a major and important challenge in the future.

## Supplementary Material

Supplemental Material
