# Supplementary material for: RNAi-Based Suppressor Screens Reveal Genetic Interactions Between the CRL2LRR-1 E3-Ligase and the DNA Replication Machinery in Caenorhabditis elegans
Source: G3 (Bethesda). 2016 Aug 18;6(10):3431–42. doi: 10.1534/g3.116.033043 (PMC5068962; doi:10.1534/g3.116.033043)
Supplement: Supplemental Material [file supp_6_10_3431__index.html]

RNAi-Based Suppressor Screens Reveal Genetic Interactions Between the CRL2LRR-1 E3-Ligase and the DNA Replication Machinery in Caenorhabditis elegans — Supplemental Material 

# RNAi-Based Suppressor Screens Reveal Genetic Interactions Between the CRL2LRR-1 E3-Ligase and the DNA Replication Machinery in *Caenorhabditis elegans*

## Supplemental Material for Ossareh-Nazari, *et al*, 2016

**Files in this Data Supplement:**

- Figure S1 - Protein Sequence alignments of CDC-7 with the homologues from different species. (.tif, 25,003 KB)
- Table S1 - List of the targeted genes in the RNAi screens. (.xlsx, 17 KB)
- Table S2 - Numerical values and statistical analysis in support of Figure 2 and 5. (.xlsx, 38 KB)
