# Supplementary figures and images for: RNAi-Based Suppressor Screens Reveal Genetic Interactions Between the CRL2LRR-1 E3-Ligase and the DNA Replication Machinery in Caenorhabditis elegans
Source: G3 (Bethesda). 2016 Aug 18;6(10):3431–42. doi: 10.1534/g3.116.033043 (PMC5068962; doi:10.1534/g3.116.033043)

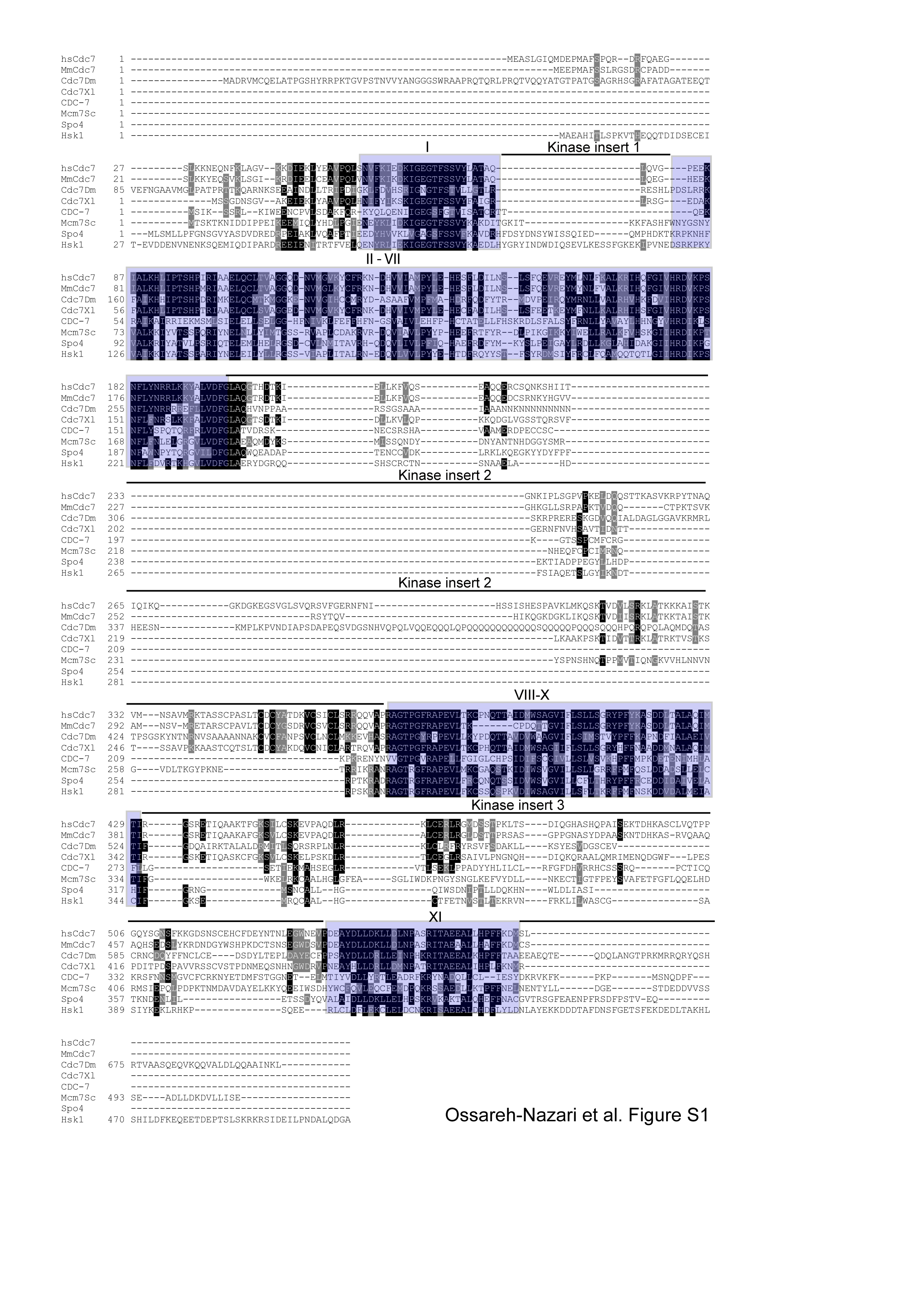

Supplement: Supplemental Material [file supp_g3.116.033043_FigureS1.tif]
